# Left Ventricular Recovery Following Bromocriptine Initiation at Different Stages of Peripartum Cardiomyopathy: A Report of Two Cases

**DOI:** 10.7759/cureus.114483

**Published:** 2026-08-13

**Authors:** Tomohide Sato, Teruhiko Ito, Ryusuke Tsunoda, Jyunjiro Koyama

**Affiliations:** 1 Division of Cardiology, Saiseikai Kumamoto Hospital Cardiovascular Center, Kumamoto, JPN; 2 Department of Cardiology, Sakurajuji Hospital, Kumamoto, Kumamoto, JPN; 3 Department of Cardiology, Japan Red Cross Kumamoto Hospital, Kumamoto, JPN

**Keywords:** bromocriptine therapy, cardiogenic shock, left ventricular recovery, peripartum cardiomyopathy, prolactin

## Abstract

Peripartum cardiomyopathy (PPCM) is a rare yet potentially life-threatening form of heart failure that occurs during late pregnancy or within the months following delivery. Bromocriptine, which suppresses prolactin secretion, has emerged as a promising adjunctive therapy for PPCM; however, the optimal timing of its initiation remains uncertain. We describe two patients with PPCM who received bromocriptine at different stages of the disease course. The first patient was a 30-year-old woman who presented 33 days after delivery with cardiogenic shock, a left ventricular ejection fraction (LVEF) of 12%, and a left ventricular thrombus. Despite treatment with intra-aortic balloon pumping, venoarterial extracorporeal membrane oxygenation, anticoagulation, and guideline-directed medical therapy, recovery of left ventricular function plateaued. Bromocriptine was initiated on hospital day 44, after which LVEF increased from 38% to 53% within seven days and subsequently recovered to 60%. The second patient was a 35-year-old woman who developed acute heart failure nine days after delivery with an LVEF of 28%. Because left ventricular function remained markedly impaired despite standard heart failure therapy, bromocriptine was initiated on hospital day seven. Her LVEF improved to 39% by hospital day 15 and ultimately recovered to 61%. These cases demonstrate a temporal association between bromocriptine initiation and subsequent improvement in left ventricular systolic function at different stages of the clinical course of PPCM.

## Introduction

Peripartum cardiomyopathy (PPCM) is a rare but potentially life-threatening form of heart failure characterized by left ventricular systolic dysfunction that develops during late pregnancy or within the months following delivery in women without preexisting structural heart disease [1]. Although its precise pathophysiology remains incompletely understood, increasing evidence implicates oxidative stress-induced cleavage of prolactin into a biologically active 16-kDa fragment as a key mediator of myocardial injury and impaired angiogenesis [2,3]. Current management of PPCM is primarily based on guideline-directed medical therapy (GDMT), adapted to the pregnancy and postpartum periods. Based on the proposed role of prolactin in the pathophysiology of PPCM, bromocriptine, a dopamine D2 receptor agonist that suppresses prolactin secretion, may be considered as an adjunctive therapy in selected patients, consistent with current European Society of Cardiology guidelines [4-7]. Randomized studies have suggested that bromocriptine may be associated with improved left ventricular systolic function in patients with PPCM; however, the available evidence remains limited [4,5]. However, the optimal timing of bromocriptine initiation remains uncertain. This issue is particularly relevant because substantial spontaneous recovery of left ventricular systolic function may occur in PPCM, especially during the early months after diagnosis, making it difficult to distinguish treatment-associated improvement from the natural course of the disease. Here, we describe the cases of two patients with PPCM who experienced substantial left ventricular recovery following bromocriptine initiation at different stages of the disease course.

## Case presentation

Case 1

A 30-year-old previously healthy primiparous woman delivered her first child vaginally at 37 weeks of gestation without perinatal complications. Bilateral lower extremity edema developed seven days after delivery, followed by loss of appetite on postpartum day 27. She was initially diagnosed with postpartum depression at a local psychiatric clinic and treated with olanzapine (0.625 mg/day) and venlafaxine (37.5 mg/day). However, her symptoms persisted. Initial laboratory testing demonstrated marked hepatic dysfunction and a markedly elevated B-type natriuretic peptide (BNP) level, and chest radiography revealed cardiomegaly. She was therefore referred to our hospital for further evaluation and treatment on postpartum day 33.

On admission, her heart rate was 163 beats/min, blood pressure was 104/74 mmHg, respiratory rate was 30 breaths/min, body temperature was 36.4°C, and oxygen saturation was 98% on room air. Physical examination revealed jugular venous distension, bilateral coarse crackles, a grade II/VI systolic murmur, third and fourth heart sounds, and bilateral lower extremity edema, consistent with severe congestive heart failure (New York Heart Association functional class IV).

Laboratory investigations revealed markedly elevated BNP (4,381.8 pg/mL), severe hepatic dysfunction (aspartate aminotransferase, 1,283 U/L; alanine aminotransferase, 1,027 U/L), mild renal dysfunction (creatinine, 1.29 mg/dL), and hyperbilirubinemia (total bilirubin, 3.0 mg/dL), consistent with systemic hypoperfusion in the setting of cardiogenic shock. Serum prolactin was 14.2 ng/mL. Thyroid function test results were within the normal range, and autoimmune serological testing and viral studies showed no significant abnormalities. Detailed laboratory findings are summarized in Table 1.

**Table 1:** Laboratory findings on admission.

Tests performed	Result	Reference range
White blood cells (/μL)	9,760	3,300-8,600
Red blood cells (×10⁴/μL)	500	386-492
Hemoglobin (g/dL)	14.6	11.6-14.8
Hematocrit (%)	44.9	35.1-44.4
Platelet count (×10⁴/μL)	28.3	15.8-34.8
Mean corpuscular volume (fL)	89.8	83.6-98.2
Mean corpuscular hemoglobin (pg)	29.2	27.5-33.2
Mean corpuscular hemoglobin concentration (%)	32.5	31.7-35.3
Albumin (g/dL)	3.3	4.1-5.1
Blood urea nitrogen (mg/dL)	32.7	8-20
Creatinine (mg/dL)	1.29	0.46-0.79
Uric acid (mg/dL)	6.5	2.6-7.0
Glucose (mg/dL)	96	73-109
Sodium (mEq/L)	132	138-145
Potassium (mEq/L)	5.3	3.6-4.8
Chloride (mEq/L)	100	101-108
Aspartate aminotransferase (U/L)	1,283	13-30
Alanine aminotransferase (U/L)	1,027	7-23
Lactate dehydrogenase (U/L)	1,384	124-222
Alkaline phosphatase (U/L)	170	38-113
Creatine kinase (U/L)	523	41-153
Total bilirubin (mg/dL)	3.0	0.4-1.5
Total cholesterol (mg/dL)	167	142-248
C-reactive protein (mg/dL)	0.58	<0.14
Hemoglobin A1c (%)	5.6	4.9-6.0
B-type natriuretic peptide (pg/mL)	4,381.8	<18.4
Prothrombin activity (%)	50	70-130
Activated partial thromboplastin time (s)	25.1	25.0-40.0
D-dimer (μg/mL)	11.2	<1.0
Thyroid-stimulating hormone (μIU/mL)	3.577	0.50-5.00
Free thyroxine (ng/dL)	1.19	0.90-1.70
Prolactin (ng/mL)	14.2	4.3-32.4
Hepatitis B surface antigen	Negative	Negative
Hepatitis B surface antibody	<3	<10
Hepatitis C virus antibody	Negative	Negative
Collagen disease-related markers	All negative	Negative

**Figure 1:**
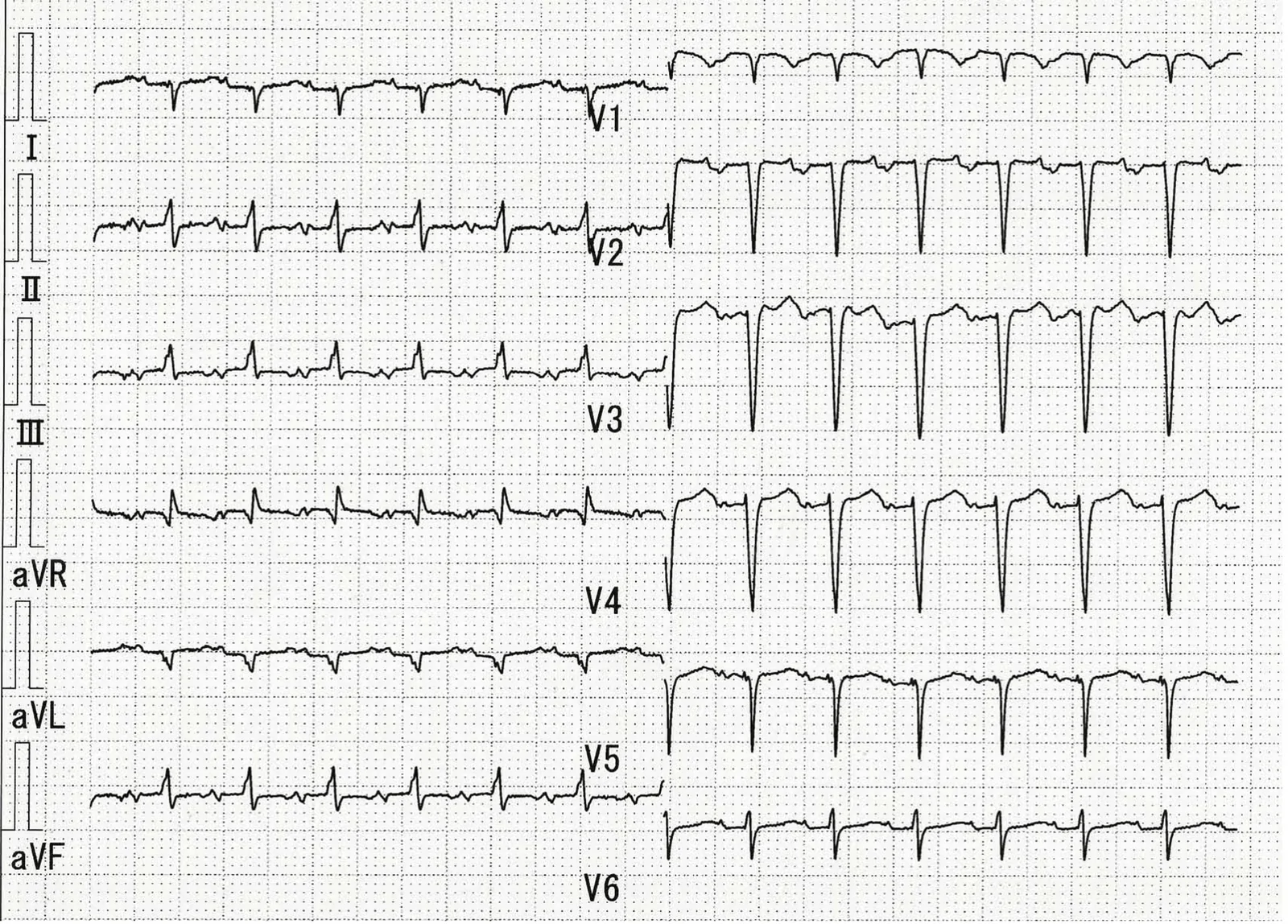
Twelve-lead electrocardiogram on admission The electrocardiogram demonstrated a narrow QRS tachycardia with a heart rate of approximately 160 beats/min, suggestive of atrial tachycardia. Negative T waves were observed in leads II, III, and aVF, together with poor R-wave progression in the precordial leads (V1–V5).

**Figure 2:**
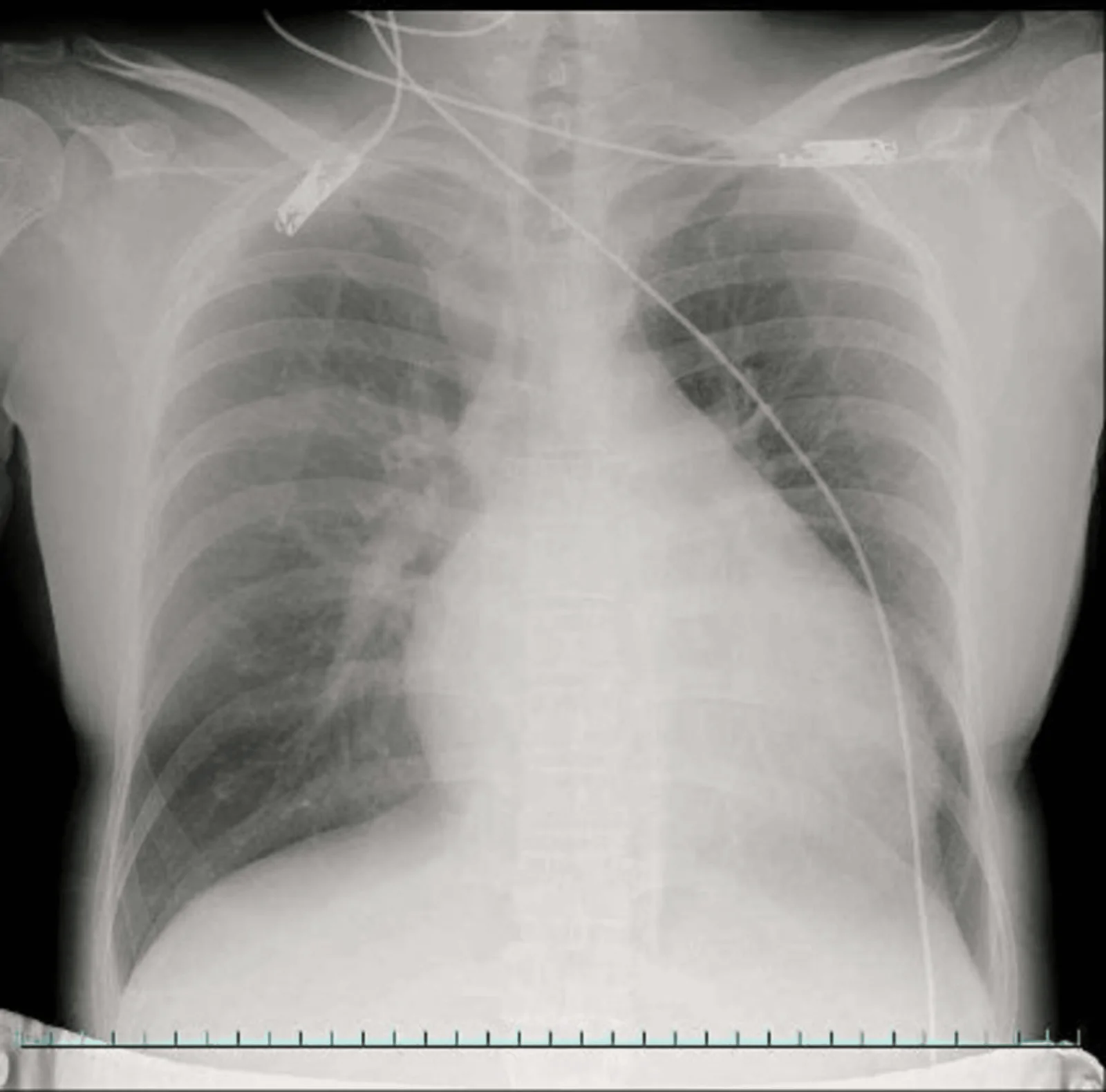
Chest radiograph on admission Chest radiography demonstrated marked cardiomegaly with a cardiothoracic ratio of 61.1% and pulmonary vascular congestion.

Electrocardiography demonstrated a narrow QRS tachycardia, suggestive of atrial tachycardia (Figure 1). Chest radiography demonstrated marked cardiomegaly with a cardiothoracic ratio of 61.1% and pulmonary vascular congestion (Figure 2). 

**Figure 3:**
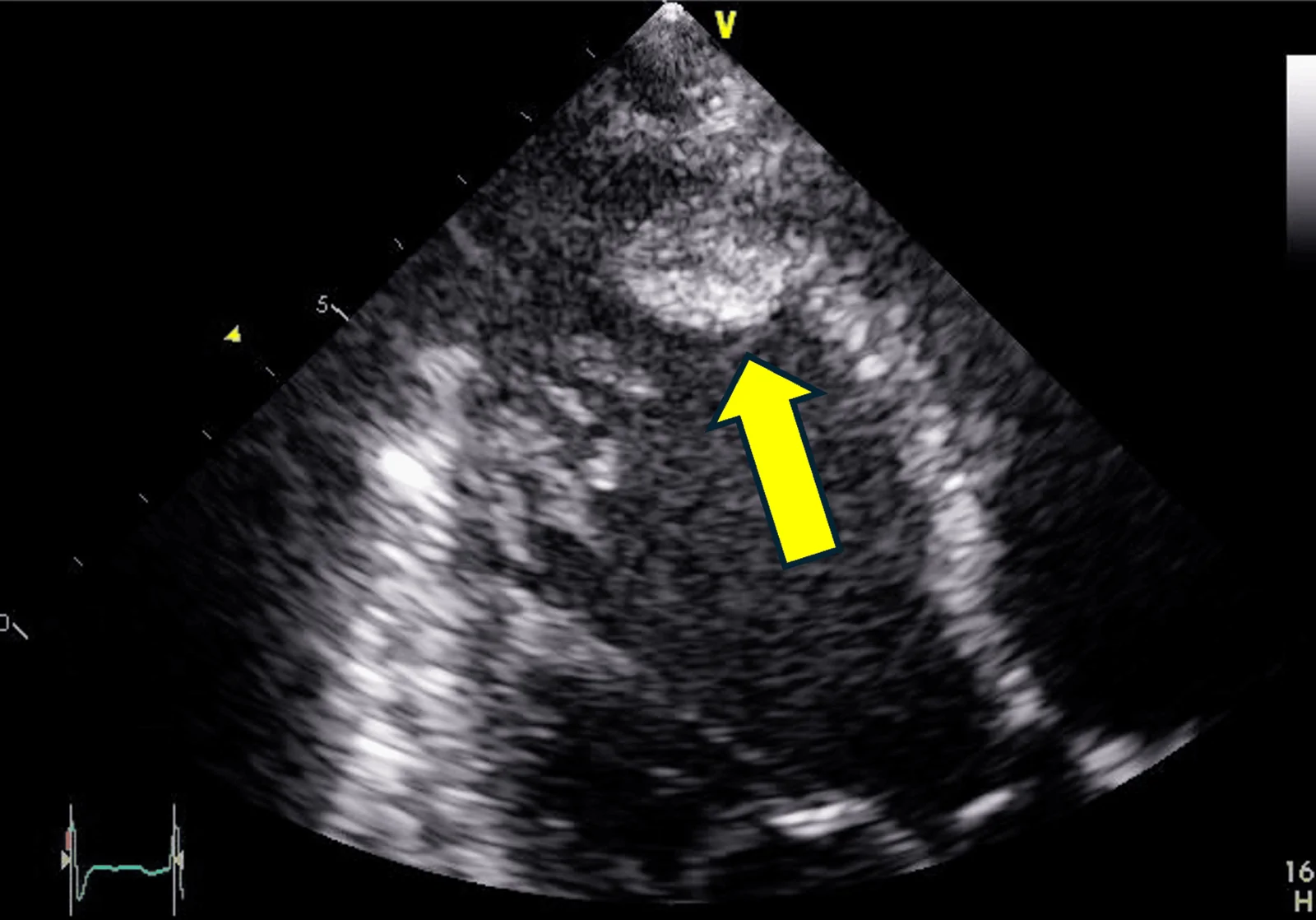
Transthoracic echocardiography on admission Apical four-chamber view demonstrating severe diffuse left ventricular systolic dysfunction with a left ventricular ejection fraction of 12%. A left ventricular thrombus attached to the apical wall is indicated by the arrow.

Transthoracic echocardiography revealed severe left ventricular systolic dysfunction with a left ventricular ejection fraction (LVEF) of 12%, assessed using the modified Simpson's biplane method, a left ventricular end-diastolic diameter of 54 mm, a left ventricular end-systolic diameter of 47 mm, moderate mitral regurgitation, mild aortic regurgitation, mild tricuspid regurgitation, and a left ventricular thrombus (Figure 3).

**Figure 4:**
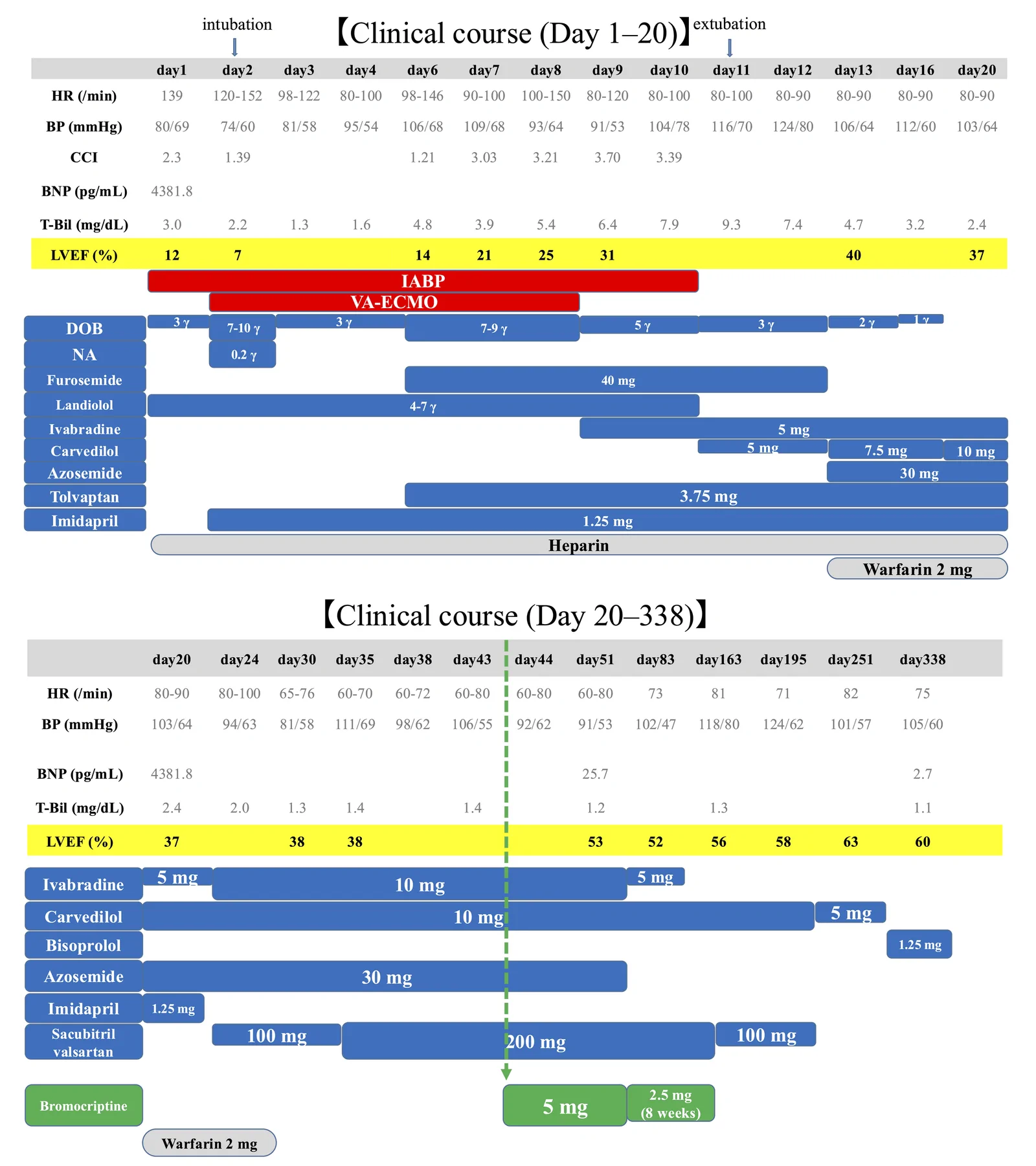
Clinical course of Case 1 Clinical course of Case 1 during hospitalization (Day 1-20): The patient presented with cardiogenic shock and required mechanical circulatory support with intra-aortic balloon pumping (IABP) and venoarterial extracorporeal membrane oxygenation (VA-ECMO). Guideline-directed medical therapy for heart failure was gradually initiated after hemodynamic stabilization. Heart rate (HR), blood pressure (BP), cardiac index (CCI), B-type natriuretic peptide (BNP), total bilirubin (T-Bil), left ventricular ejection fraction (LVEF), and major therapeutic interventions during hospitalization are shown. Serial LVEF values are highlighted in yellow. Clinical course of Case 1 after hospital day 20 (Day 20-338): Despite guideline-directed medical therapy, LVEF plateaued at approximately 37-38%. Bromocriptine was initiated on hospital day 44, after which LVEF gradually improved to 60% during follow-up. HR, BP, BNP, T-Bil, and changes in heart failure medications are summarized. Serial LVEF values are highlighted in yellow. The green dashed line indicates the initiation of bromocriptine therapy on hospital day 44. DOB: dobutamine; NA: noradrenaline.

Pulmonary embolism was considered clinically unlikely because pulmonary vascular congestion was present without right-sided cardiac enlargement on echocardiography. Coronary angiography demonstrated no significant coronary artery stenosis or findings suggestive of spontaneous coronary artery dissection. Endomyocardial biopsy revealed no findings suggestive of active myocarditis or metabolic disease. She had no history of hypertensive disorders during pregnancy or postpartum hypertension, and her blood pressure on admission was 104/74 mmHg. She had no history of cardiac disease or family history of cardiomyopathy or sudden cardiac death, and left ventricular systolic function had been normal on echocardiography performed during pregnancy. Although sinus tachycardia and atrial tachycardia were observed during the early phase of hospitalization, left ventricular systolic dysfunction persisted after heart rate control, with LVEF remaining in the 30% range despite a reduction in heart rate to approximately 60-70 beats/min (Figure 4). 

Based on the development of heart failure during the postpartum period, an LVEF <45%, and the absence of another identifiable cause of left ventricular systolic dysfunction, the patient was diagnosed with PPCM complicated by cardiogenic shock and a left ventricular thrombus [1].

Intra-aortic balloon pumping was initiated immediately; however, progressive hemodynamic deterioration necessitated venoarterial extracorporeal membrane oxygenation the following day. Intravenous catecholamines, diuretics, and GDMT, including an angiotensin-converting enzyme inhibitor and a beta-blocker, were subsequently introduced as tolerated. Persistent sinus tachycardia was managed with landiolol and ivabradine. The patient was successfully weaned from venoarterial extracorporeal membrane oxygenation on hospital day eight and extubated on hospital day 11. Follow-up echocardiography demonstrated resolution of the left ventricular thrombus. Anticoagulation was initiated on hospital day one with intravenous unfractionated heparin because of the left ventricular thrombus, with the dose adjusted according to activated partial thromboplastin time and/or activated clotting time. Anticoagulation was subsequently transitioned to warfarin and discontinued on hospital day 24 after resolution of the left ventricular thrombus. Thus, anticoagulation was not continued during bromocriptine therapy, which was initiated on hospital day 44. Although LVEF initially improved, serial echocardiography showed that it subsequently remained approximately 37-38%, with values of 37% on hospital day 20 and 38% on hospital days 30 and 35 despite continued heart failure therapy. Bromocriptine was therefore initiated on hospital day 44 at a dose of 5 mg/day for two weeks, followed by 2.5 mg/day for six weeks. LVEF subsequently increased to 53% on hospital day 51, seven days after bromocriptine initiation, and remained preserved during follow-up, reaching 63% on hospital day 251 and 60% on hospital day 338. The patient’s clinical course is summarized in Figure 4.

Case 2

A 35-year-old woman with no previous history of cardiovascular disease presented with acute dyspnea nine days after the delivery of her first child at 41 weeks of gestation. Dyspnea on exertion had developed eight days postpartum and rapidly progressed. She visited a local clinic, where her peripheral oxygen saturation was 78% on room air, prompting emergency transfer to our hospital. She had no significant medical history and was not taking any regular medications.

On admission, the patient was alert. Her body temperature was 38.3°C, blood pressure was 141/111 mmHg, heart rate was 156 beats/min, respiratory rate was 46 breaths/min, and oxygen saturation was 78% despite oxygen administration at 10 L/min. Physical examination revealed bilateral coarse crackles, lower extremity edema, and third and fourth heart sounds, consistent with acute decompensated heart failure.

Laboratory investigations demonstrated markedly elevated BNP (1,882.3 pg/mL) levels, anemia (hemoglobin, 9.0 g/dL), leukocytosis (white blood cell count, 23,340/μL), elevated C-reactive protein (5.71 mg/dL) levels, and elevated D-dimer (4.8 μg/mL) levels. Urine culture was positive for Escherichia coli, whereas blood cultures were negative. The patient was treated with ceftriaxone for 10 days, with gradual resolution of fever and decreases in the white blood cell count and C-reactive protein level. Thyroid function was normal (thyroid-stimulating hormone, 1.044 μU/mL; free thyroxine, 0.88 ng/dL). Serum prolactin concentration was 118 ng/mL. Serologic testing for hepatitis B surface antigen and hepatitis C antibody was negative. Detailed laboratory findings are summarized in Table 2.

**Table 2:** Laboratory findings on admission

Tests performed	Result	Reference range
White blood cells (/μL)	23,340	3,300-8,600
Red blood cells (×10⁴/μL)	303	386-492
Hemoglobin (g/dL)	9.0	11.6-14.8
Hematocrit (%)	29.9	35.1-44.4
Platelet count (×10⁴/μL)	38.1	15.8-34.8
Mean corpuscular volume (fL)	98.7	83.6-98.2
Mean corpuscular hemoglobin (pg)	29.7	27.5-33.2
Mean corpuscular hemoglobin concentration (%)	30.1	31.7-35.3
Albumin (g/dL)	2.4	4.1-5.1
Blood urea nitrogen (mg/dL)	7.5	8-20
Creatinine (mg/dL)	0.52	0.46-0.79
Glucose (mg/dL)	142	73-109
Sodium (mEq/L)	139	138-145
Potassium (mEq/L)	3.8	3.6-4.8
Chloride (mEq/L)	104	101-108
Aspartate aminotransferase (U/L)	27	13-30
Alanine aminotransferase (U/L)	19	7-23
Lactate dehydrogenase (U/L)	315	124-222
Alkaline phosphatase (U/L)	86	38-113
Creatine kinase (U/L)	283	41-153
Total bilirubin (mg/dL)	0.2	0.4-1.5
Hemoglobin A1c (%)	5.3	4.9-6.0
C-reactive protein (mg/dL)	5.71	<0.14
B-type natriuretic peptide (pg/mL)	1,882.3	<18.4
Prothrombin activity (%)	80	70-130
Activated partial thromboplastin time (s)	26.3	25.0-40.0
D-dimer (μg/mL)	4.8	<1.0
Thyroid-stimulating hormone (μIU/mL)	1.044	0.50-5.00
Free thyroxine (ng/dL)	0.88	0.90-1.70
Prolactin (ng/mL)	118	4.3-32.4
Hepatitis B surface antigen	Negative	Negative
Hepatitis C virus antibody	Negative	Negative

**Figure 5:**
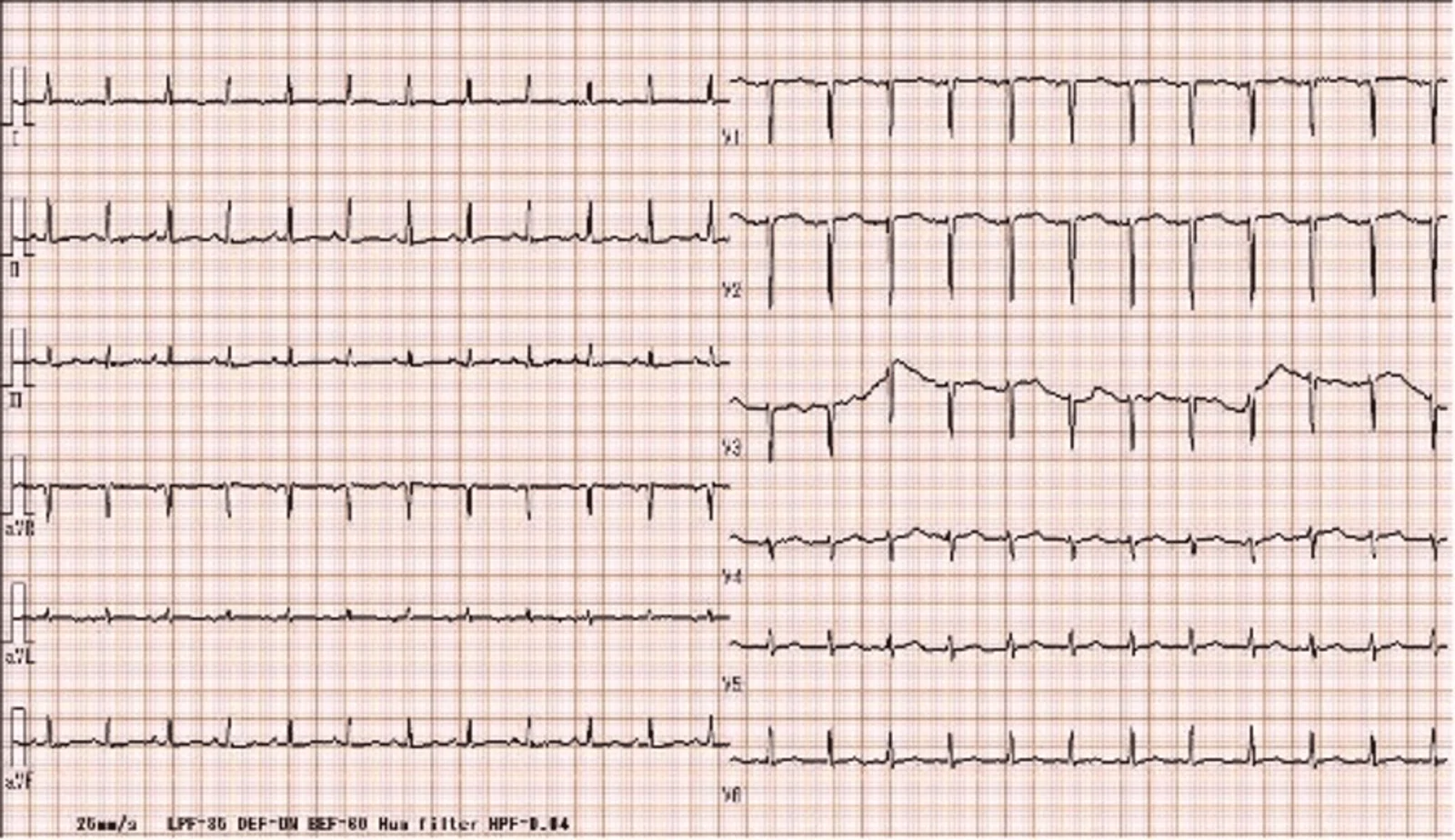
Twelve-lead electrocardiogram on admission The electrocardiogram demonstrated sinus tachycardia with a heart rate of approximately 140 beats/min together with poor R-wave progression in the precordial leads (V1–V4).

**Figure 6:**
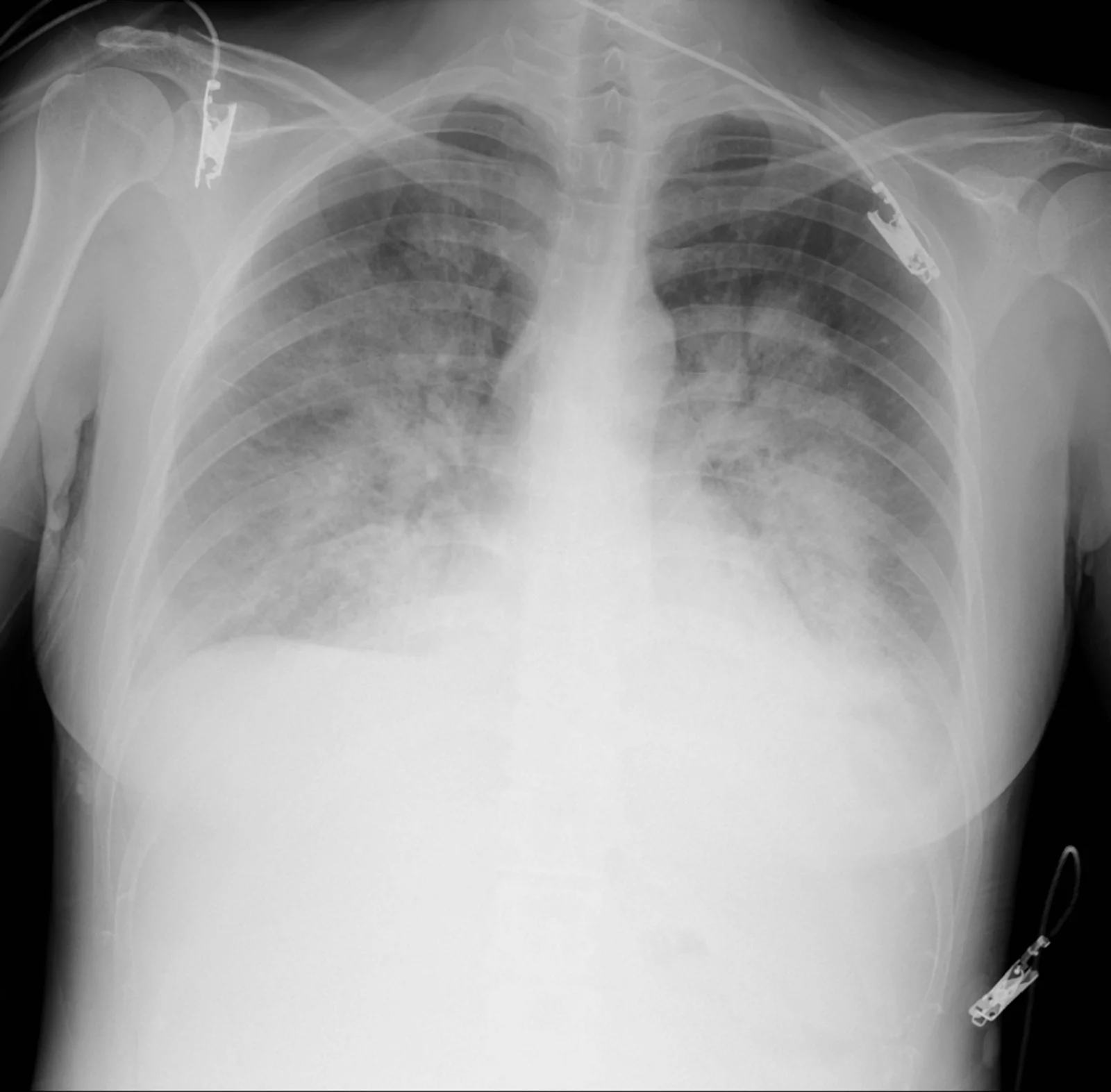
Chest radiograph on admission Chest radiography demonstrated cardiomegaly with a cardiothoracic ratio of 52% and pulmonary vascular congestion.

A 12-lead electrocardiogram demonstrated sinus tachycardia (heart rate, 140 beats/min) with poor R-wave progression in leads V1-V4 (Figure 5). Chest radiography demonstrated cardiomegaly with a cardiothoracic ratio of 52% and pulmonary vascular congestion (Figure 6).

**Figure 7:**
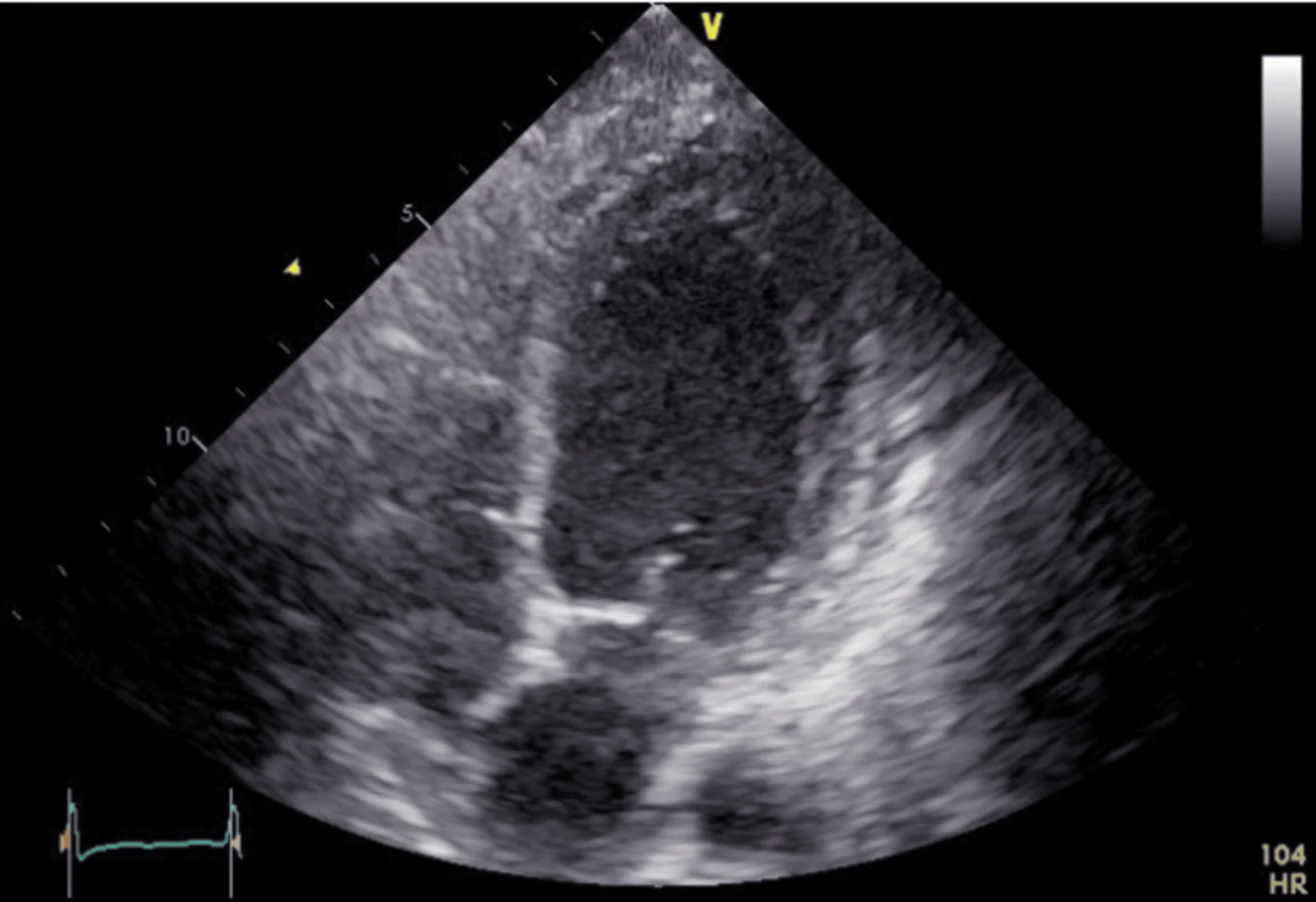
Transthoracic echocardiography on admission Transthoracic echocardiography demonstrated severe diffuse left ventricular systolic dysfunction with a left ventricular ejection fraction of 28%. No left ventricular thrombus was observed.

Transthoracic echocardiography revealed diffuse left ventricular systolic dysfunction with an LVEF of 28%, assessed using the modified Simpson's biplane method, a left ventricular end-diastolic diameter of 45.1 mm, and a left ventricular end-systolic diameter of 39.3 mm. Mild mitral regurgitation was present, and no left ventricular thrombus was observed (Figure 7).

**Figure 8:**
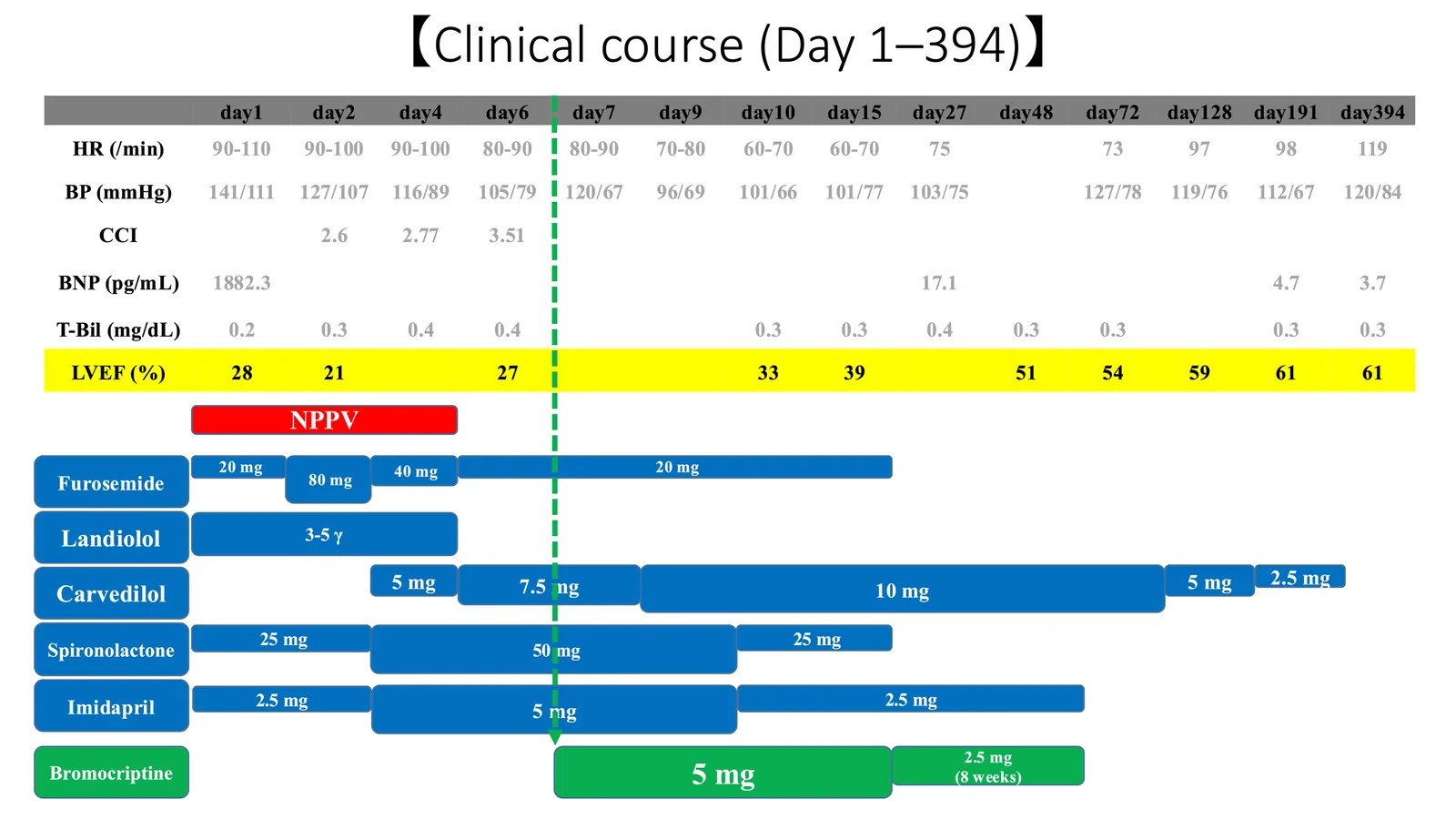
Clinical course of Case 2 Guideline-directed medical therapy was initiated immediately after admission. Because left ventricular systolic dysfunction persisted despite standard heart failure treatment, bromocriptine was started on hospital day 7. Left ventricular ejection fraction (LVEF) gradually improved from 28% at admission to 39% on hospital day 15 and ultimately recovered to 61% during follow-up. Serial LVEF values are highlighted in yellow. The green dashed line indicates the initiation of bromocriptine therapy on hospital day 7. HR: heart rate, BP: blood pressure, CCI: cardiac index, BNP: B-type natriuretic peptide, T-Bil: total bilirubin

Pulmonary embolism was considered clinically unlikely because pulmonary vascular congestion was present without right-sided cardiac enlargement on echocardiography. Coronary angiography demonstrated no significant coronary artery stenosis or findings suggestive of spontaneous coronary artery dissection. Endomyocardial biopsy revealed no findings suggestive of active myocarditis or metabolic disease. She had no history of hypertensive disorders of pregnancy, including preeclampsia, and no hypertension had been documented during the postpartum period before the onset of heart failure. Although her blood pressure was elevated to 141/111 mmHg on admission, it subsequently stabilized without persistent hypertension during hospitalization (Figure 8). She had no history of cardiac disease or family history of cardiomyopathy or sudden cardiac death, and left ventricular systolic function had been normal on echocardiography performed during pregnancy. Although sinus tachycardia was present on admission, left ventricular systolic dysfunction persisted after heart rate control, with LVEF remaining in the 20% range despite a reduction in heart rate to approximately 80-90 beats/min (Figure 8). Based on the development of heart failure during the postpartum period, an LVEF <45%, and the absence of another identifiable cause of left ventricular systolic dysfunction, the patient was diagnosed with PPCM [1].

Non-invasive positive-pressure ventilation was initiated immediately. GDMT, including an angiotensin-converting enzyme inhibitor, a beta-blocker, spironolactone, and diuretics, was subsequently introduced. No left ventricular thrombus was detected, and anticoagulation therapy was not administered before or during bromocriptine treatment. However, serial echocardiography demonstrated persistent severe left ventricular systolic dysfunction, with LVEF values of 28% on hospital day one, 21% on day two, and 27% on day six. Bromocriptine was therefore initiated on hospital day seven at a dose of 5 mg/day for two weeks, followed by 2.5 mg/day for six weeks. LVEF subsequently increased to 33% on hospital day 10 and 39% on day 15, three and eight days after bromocriptine initiation, respectively. LVEF further increased to 51% on hospital day 48 and remained preserved during long-term follow-up, reaching 61% on hospital day 191 and remaining at 61% on hospital day 394. Heart failure medications were gradually tapered without recurrence of left ventricular systolic dysfunction. The patient’s clinical course is summarized in Figure 8.

## Discussion

Despite recent advances in the understanding of PPCM, several important clinical questions remain unresolved, including the optimal timing of bromocriptine initiation. Although GDMT remains the cornerstone of treatment, accumulating clinical evidence over the past two decades suggests that bromocriptine may provide additional therapeutic benefit as an adjunctive therapy in selected patients with PPCM [1-5]. However, robust evidence regarding the optimal timing of bromocriptine initiation remains lacking, particularly in patients whose recovery has plateaued despite optimized GDMT.

The rationale for bromocriptine therapy is supported by both experimental and clinical evidence. Experimental studies have demonstrated that oxidative stress-induced cleavage of the native 23-kDa prolactin into a biologically active 16-kDa fragment, as well as the imbalance in angiogenesis, play important roles in the pathogenesis of PPCM [2,3]. Consistent with these findings, a pilot randomized study by Sliwa et al. demonstrated that adjunctive bromocriptine therapy was associated with greater improvement in left ventricular systolic function than standard heart failure therapy alone [4]. In addition, the multicenter randomized study by Hilfiker-Kleiner et al. reported substantial improvement in left ventricular function with both short- and long-term bromocriptine treatment; however, prolonged therapy was associated with a higher rate of complete recovery in patients with severe PPCM [5]. Based on these findings, the Heart Failure Association of the European Society of Cardiology recommended that bromocriptine may be considered in selected patients with PPCM, particularly in severe cases, provided that appropriate anticoagulation is administered [6]. Against this background, the present cases provide an opportunity to explore the potential clinical implications of bromocriptine initiation at different stages of the disease course.

These cases highlight that PPCM should be considered in postpartum women presenting with new-onset heart failure symptoms, particularly when accompanied by tachycardia, elevated natriuretic peptide levels, and otherwise unexplained left ventricular systolic dysfunction [1]. The present cases highlight the potential clinical implications of bromocriptine initiation at different stages of the disease course, an issue that has received little attention in previous reports. Although the timing of bromocriptine initiation differed substantially between the two patients, both experienced improvement in left ventricular systolic function following treatment. In Case 1, bromocriptine was introduced only after prolonged intensive care, mechanical circulatory support, and GDMT optimization, because left ventricular function had plateaued. In contrast, Case 2 received bromocriptine relatively early during hospitalization because severe left ventricular dysfunction persisted despite initial heart failure therapy. Despite these differences in disease severity and treatment timing, both patients ultimately demonstrated normalization of left ventricular systolic function. Because spontaneous recovery is well recognized in PPCM, it cannot be determined with certainty whether bromocriptine contributed to the observed recovery in these two patients. Nevertheless, the temporal association between bromocriptine initiation and subsequent improvement in left ventricular systolic function raises the possibility that bromocriptine may have contributed to further improvement beyond that achieved with GDMT alone. Further prospective studies are warranted to investigate the timing, duration, and patient selection for bromocriptine therapy in patients with PPCM.

This report has several limitations. First, it describes only two patients, precluding any definitive conclusions regarding the efficacy or optimal timing of bromocriptine therapy. Second, because this is a case report, and spontaneous recovery is well recognized in PPCM, a causal relationship between bromocriptine therapy and left ventricular recovery cannot be established. In addition, LVEF measurements may have been influenced by changes in loading conditions and other concomitant therapies during the clinical course.

## Conclusions

We reported two patients with PPCM who experienced substantial recovery of left ventricular systolic function following bromocriptine therapy initiated at different stages of the disease course. These cases suggest a potential association between bromocriptine initiation and subsequent improvement in left ventricular systolic function, even when treatment is introduced after an apparent plateau in recovery with GDMT. These observations support further prospective investigation of the timing, duration, and patient selection for bromocriptine therapy in PPCM.
